# Epidemiology of urinary tract infections and antibiotics sensitivity among pregnant women at Khartoum North Hospital

**DOI:** 10.1186/1476-0711-10-2

**Published:** 2011-01-18

**Authors:** Hamdan Z Hamdan, Abdel Haliem M Ziad, Salah K Ali, Ishag Adam

**Affiliations:** 1 Faculty of Medicine, Al-Neelain University, Khartoum, Sudan; 2 Khartoum North Hospital, Khartoum, Sudan; 3 National health laboratory, Khartoum, Sudan; 4 Faculty of Medicine, University of Khartoum, Khartoum, Sudan

## Abstract

**Background:**

Urinary tract infections (UTI) can lead to poor maternal and perinatal outcomes. Investigating epidemiology of UTI and antibiotics sensitivity among pregnant women is fundamental for care-givers and health planners.

**Methods:**

A cross sectional study has been conducted at Khartoum north teaching hospital Antenatal Care Clinic between February-June 2010, to investigate epidemiology of UTI and antibiotics resistance among pregnant women. Structured questionnaires were used to gather data from pregnant women. UTI was diagnosed using mid stream urine culture on standard culture media

**Results:**

Out of 235 pregnant women included, 66 (28.0%) were symptomatic and 169 (71.9%) asymptomatic. the prevalence of bacteriuria among symptomatic and asymptomatic pregnant women were (12.1%), and (14.7%) respectively, with no significant difference between the two groups (*P *= 0.596), and the overall prevalence of UTI was (14.0%). In multivariate analyses, age, gestational age, parity, and history of UTI in index pregnancy were not associated with bacteriuria. *Escherichia coli *(42.4%) and *S. aureus *(39.3%) were the commonest isolated bacteria. Four, 2, 2, 3, 4, 2 and 0 out of 14 *E. coli *isolates, showed resistance to amoxicillin, naladixic acid, nitrofurantoin, ciprofloxacin, co-trimoxazole, amoxicillin/clavulanate and norfloxacin, respectively

**Conclusion:**

*Escherichia coli *were the most prevalent causative organisms and showing multi drug resistance pattern, asymptomatic bacteriuria is more prevalent than symptomatic among pregnant women. Urine culture for screening and diagnosis purpose for all pregnant is recommended.

## Introduction

Due to several anatomical and hormonal changes, pregnant women are more susceptible to develop Urinary tract infections (UTI) [1]. UTI is a major health problem, it has been reported among 20% of the pregnant women and it is the most common cause of admission in obstetrical wards [2]. Symptomatic and asymptomatic bacteriuria have been reported among 17.9% and 13.0% pregnant women, respectively [3].

UTI (perhaps if untreated) can lead to serious obstetric complications, poor maternal and perinatal outcomes e.g. intrauterine growth restriction, pre-eclampsia, caesarean delivery and preterm deliveries [4]. Furthermore, it has been observed that asymptomatic bacteriuria can lead to cystitis and pyelonephritis [5] which can lead to acute respiratory distress, transient renal failure, sepsis and shock during pregnancy [6]. Screening of pregnant women for UTI can minimize these UTI associated complications [7]. Recently various risk factors of UTI during pregnancy have been reported; perhaps these are varied according the geographical, social and biological settings [8]. *Escherichia coli*- with its multidrug resistant strains- has been found to be the commonest cause of UTI among pregnant women [9,10].

Investigating epidemiology of UTI (prevalence, risk factors, bacterial isolates and antibiotic sensitivity) during pregnancy is fundamental for care givers and health planners to guide the expected interventions. While an extensive published literature concerning UTI during pregnancy is available from other African countries [11], there is no published data concerning UTI in pregnant Sudanese women. Thus, this was the objective of this study which has been conducted at the Antenatal Care Clinic of Khartoum North hospital during the period of February-June 2010.

## Methods

A cross-sectional study has been conducted at Khartoum North hospital Antenatal Care Clinic during the period of February-June 2010. Consecutive pregnant women who attended the Antenatal Care Clinic for the first time was approached to participate in the study. Those with known underline renal pathology or chronic renal disease were excluded. After signing an informed consent, relevant medical, obstetrical and socio-demographic characteristics were gathered using pre-tested questionnaires. Every woman was inquired for history suggestive of UTI (urgency, frequency, loin pain etc) and history of using antibiotics in the index pregnancy. Maternal weight, height, and body mass index (BMI) was calculated as weight in kilograms divided by height in meters squared. Maternal haemoglobin was measured.

Mid stream urine samples were collected using sterile container on the same day of enrolment. All the specimens were analyzed within an hour of collection using dipstick (Mannheim GmbH, Germany) following manufacturer's instructions, then samples were analyzed for culture and sensitivity. By Using standard quantitative loop a 1 μl and 10 μl were used to inoculate urine sample on Cysteine lactose electrolyte deficient Agar, MacConkey and Blood agar plates (OXOID-England). Plates were incubated for 24 hr at 37°C. A diagnosis of UTI was made when there were at least 10^5 ^colony forming unit (CFU)/ml of urine. For contaminated specimens, repeat culture was performed. Identification was done using in house biochemical testing [12]. *S. aureus *was identified by colonial morphology, gram positive staining, positive catalase activity, and positive coagulation of citrated rabbit plasma (bioMe 'rieux, Marcyl'Etoile, France). Disc diffusion method was used to determine susceptibility of the isolates as previously described [13]. Individual colonies were suspended in normal saline to 0.5 McFarland and using sterile swabs the suspensions were inoculated on Muller Hinton agar for 18-24 hr. E. coli ATCC 25922 was used as control strains [14].

For gram-negative and positive bacteria the following discs were tested: amoxicillin (25 μg), co-trimoxazole (SXT) (1.25/23.75 μg), nitrofurantoin (300 μg), ciprofloxacin (5 μg), nalidixic acid (30 μg), amoxicillin-clavulanic acid (20 μg/10 μg), and norfloxacin (5 μg), Symptomatic patients were given amoxicillin/clavulanate as empirical treatment before culture results. All patients were asked to come back for results after 2 days. Then patients care at Antenatal Care Clinic has been continued by her managing obstetrician in the particular unit.

### Statistics

Data were entered in the computer using SPSS for windows version13.0 and double checked before analysis. Means and proportions of the socio-demographic and obstetrical characteristics were calculated and compared between the growth positive and negative groups using student *t *and  X^2^ tests, respectively. Univariate and multivariate analysis were used with isolate positive group as dependent variable and socio-demographic and obstetrics variables as independent variables. Probability values of <0.05 were considered as statistically significant for all results.

## Results

Two hundred and thirty-five pregnant women were enrolled at the mean (SD) gestational age of 29 (7.9) weeks. The mean (SD) of their age and parity were 27.5 (14.6) years and 2.6 (2.4), respectively. Out of 235 pregnant women, 66 (28.0%) had symptoms suggestive of UTI. The prevalence of bacteriuria among symptomatic and asymptomatic pregnant women were (12.1%), and (14.7%), respectively, with no significant difference between the two groups, and the overall prevalence of UTI was (14.0%). Interestingly out of 33 who had significant bacteriuria, 14 (42.2%) had a history of UTI in current pregnancy and received antibiotic for that UTI. There was no significant difference in the socio-demographic and clinical data between bacteriuric and abacteriuric women, table 1.

**Table 1:** Obstetrical characteristic between bacteriuric and abacteriuric women in Khartoum north hospital, Sudan.

Variables	Women with Bacteriuria	Women without Bacteriuria	*P*
Age, years	25.7(5.3)	27.8(15.6)	0.438
Parity	2(2.1)	2.7(2.5)	0.132
Gestational Age, weeks	29.6(6.9)	29.2(8.1)	0.783
Weight, Kg	65.8(9)	67.8(7.1)	0.245
Height, meter	1.6(6.)	1.6(6.)	0.007
Body mass index	24.8(2.7)	24.7(2.5)	0.845
History of UTI	14(42.4)	95(47)	0.623
History of Antibiotic use	14(42.4)	89(44)	0.861
Hemoglobin, g/dl	9.7 (.9)	9.7 (0.9)	0.901
Dysuria	8(24.2)	58(28.7)	0.596
Urgency	1(3)	5(2.5)	1.000
Fever	33(100)	2(1)	1.000
Vomiting	33(100)	2(1)	1.000

Data were shown as mean (SD) or n (%) as applicable.

### Risk factors of urinary tract infections

None of the investigated factors (age, gestational age, parity, symptoms and body mass index) were found as risk factor for UTI in univariate and multivariate analysis, table 2.

**Table 2:** Factors associated with UTI in pregnancy in Khartoum North Hospital, Sudan using univariate and multivariate analyses.

Variables	Univariate analysis			Multivariate analysis		
	**OR**	**95%CI**	***P***	**OR**	**95%CI**	***P***

Age	0.9	0.8-1.0	0.2	0.9	0.8-1.0	0.2
Body mass index	1.0	0.8-1.1	0.8	1.0	0.8-1.2	0.4
Parity	0.8	0.7-1.0	0.1	0.9	0.7-1.1	0.4
Gestational age	1.0	0.9-1.0	0.7	1.0	0.9-1.0	0.9
Dysuria	0.7	0.3-1.8	0.5	0.8	0.3-2.5	0.8
Urgency	1.2	0.1-10.8	0.8	1.9	0.1-22.1	0.5
History of UTI	0.8	0.3-1.7	0.6	0.3	0.1-6.6	0.4
History of antibiotic use	0.9	0.4-1.9	0.8	3.0	0.1-64.9	0.4

Abbreviations: OR, Odds Ratio; CI, confidence interval

### Bacterial isolates and their sensitivity

Eighteen (54.5%) and 15 (45.4%) of the 33 isolates were gram negative and positive bacteria, respectively. *E. coli *[14 (42.4%)] was the most predominant organism isolated. Other isolates were *S. aureus *[13 (39.3%)], *K. pneumoniae *[3 (9%)], *group B streptococcus *[2 (6%)] and *P. aeruginosa *[1 (3%)].

Four, 2, 2, 3, 4, 2 and 0 out of 14 *E. coli *isolates, showed resistance to amoxicillin, naladixic acid, nitrofurantoin, ciprofloxacin, co-trimoxazole, amoxicillin/clavulanate and norfloxacin, respectively. Thirteen *S. aureus *isoltes showed resistant to amoxicillin (1), norfloxacin (3), co-trimoxazole (5), and naladixic acid (5). *K. pneumonia *isolates (3) have resistance to amoxicillin (2), both naladixic acid and amoxicillin/clavulanate (1). There was no resistance to co-trimoxazole, nitrofurantoin, norfloxacin and ciprofloxacin. One of the two *group B streptococcus *isolates has resistance to naladixic acid while sensitive to amoxicillin, nitrofurantoin, amoxicillin/clavulanate, norfloxacin, co-trimoxazole and ciprofloxacin. One *P. aeruginosa *isolate has resistance to amoxicillin, nitrofurantoin, and co-trimoxazole, while sensitive to naladixic acid, ciprofloxacin, amoxicillin/clavulanate, norfloxacin.

Out of 33 who had positive culture growth 4 had a nitrate test positive, while 202 who had no growth in the culture media only one had a false positive nitrate test, this make the sensitivity and specificity of the nitrate test versus culture growth as 12.1% and 99.5% respectively.

## Discussion

The main findings of this study were: the prevalence of UTI among pregnant women was 14.0% - regardless to the women's age, parity and gestational age -and *E. coli *was the commonest isolated organism with multi resistance toward different antibiotics. The prevalence of UTI among these women is similar to the prevalence of UTI among pregnant women in the neighbor countries e.g. 14.6% and 11.6% in Tanzania and Ethiopia [3,11].

Age, parity and gestational age were not associated with UTI in this study as well as in neighboring Tanzania [3]. However, maternal age, parity and morbid obesity have been previously observed as risk factors for UTI among pregnant women [8,15,16]. Likewise in this study gestational age was not found as risk factor for UTI among these women. Recently, it has been reported that, UTI developed in third trimester [17]. Perhaps the susceptibility of UTI during this period is due to uretral dilatation which started as early as 6 week and reaching the maximum during 22-24 weeks [9].

Other factors like low socio-economic status, sexual activity, washing genitals precoitus, postcoitus, not voiding urine postcoitus and washing genitals from back to front have observed as risk factors for UTI during pregnancy [15,18]. These factors have not been investigated in the current study; otherwise the results would have been changed. According to the traditions in central Sudan, it might have been difficult to enquire about washing genitalia and sexual activity; otherwise patients' co-operation would be lost. Interestingly high prevalence of urinary tract infection has been reported among Sudanese females with genital mutilation [19], which was widely practiced in Central Sudan [20].

In this study *E. coli was *the most common pathogen (77.7% of the Gram-negative isolates, 42.4% of all isolate). This goes with results that obtained in Tanzania where *E. coli *was 38% of the Gram-negative isolates and 25% of all isolate [21]. Likewise, many authors have the same findings e.g. in Pakistan and India [8,22]. In this study *E. coli *showed multidrug resistance mainly to amoxicillin, co-trimoxazole and nitrofurantoin. In Africa e.g. Tanzania, Kenya and Senegal it have been reported that, *E. coli *in urinary isolates have a high antimicrobial resistance pattern [3,10,23]. Likewise Gales et al and Williams et al have reported high resistance of *E. coli *towards different antimicrobials in Latin American and Costa Rica, respectively [24,25]. Although, *S. aureus *was known for years as rare urinary isolate [26], recently it has been reported to be the most frequent pathogen among pregnant women in Nigeria [27]. In this setting it was found the second most prevalent bacteria, this is in concert to the other previous observation [8].

In this study, 42.4% women who had positive isolate received an antibiotic in the index pregnancy. It has been shown that anti-microbial resistance to one drug does not always correlate to the consumption of the same drug or closely related drugs [28]. Inappropriate antimicrobial use can lead to inadequate therapy and contribute to further drug resistance [29]. The inappropriate use of antimicrobial in low income countries is perhaps due to the lack of adequate knowledge about drugs and non-availability or non-accessibility of guidelines for therapy [22] or to the availability of antimicrobials without prescription and perhaps it was prescribed by non-skilled practitioners [30].

## Conclusion

There was high prevalence of asymptomatic bacteriuria among pregnant women in this setting regardless to women's age, parity and gestational age. *E. coli *with its multi resistance towards antibiotics was the most common isolated organism. Thus urine culture should be performed as screening and diagnostic tool of UTI in pregnancy in this setting.

## Ethics

This study was approved by Sudan Medical specialization Ethics Review Board, Sudan.

## Competing interests

The authors declare that they have no competing interests.

## Authors' contributions

HZA and AMZ carried out the study and participated in the statistical analysis and procedures. IA coordinated and participated in the design of the study, statistical analysis and the drafting of the manuscript. All the authors read and approved the final version.
